# Cellular and Molecular Adaptation of Arabian Camel to Heat Stress

**DOI:** 10.3389/fgene.2019.00588

**Published:** 2019-06-19

**Authors:** Abdullah Hoter, Sandra Rizk, Hassan Y. Naim

**Affiliations:** ^1^Department of Biochemistry and Chemistry of Nutrition, Faculty of Veterinary Medicine, Cairo University, Giza, Egypt; ^2^Department of Physiological Chemistry, University of Veterinary Medicine Hannover, Hanover, Germany; ^3^School of Arts and Sciences, Lebanese American University, Beirut, Lebanon

**Keywords:** Arabian camel, heat shock proteins, heat stress, chaperones, desert, adaptation

## Abstract

To cope with the extreme heat stress and drought of the desert, the Arabian camel (*Camelus dromedarius*) has developed exceptional physiological and biochemical particularities. Previous reports focused mainly on the physiological features of Arabian camel and neglected its cellular and molecular characteristics. Heat shock proteins are suggested to play a key role in the protein homeostasis and thermotolerance. Therefore, we aim by this review to elucidate the implication of camel HSPs in its physiological adaptation to heat stress and compare them with HSPs in related mammalian species. Correlation of these molecules to the adaptive mechanisms in camel is of special importance to expand our understanding of the overall camel physiology and homeostasis.

## Introduction

Arabian camel (*Camelus dromedarius*), also known as the one humped camel, is a unique large animal belonging to the Camelidae family. This creature is well adapted to endure extreme levels of heat stress and arid conditions of the desert. Nevertheless, it has been used as a valuable source of milk, meat and wool (Kadim et al., 2008; Faye, 2015). Arabian camels exist mainly in the Middle East and parts of tropical and subtropical regions (Dorman, 1984). Historically, camels were widely used as a principal mean of transport of humans and goods between countries, hence known as the ship of desert (Bornstein, 1990). Recently, additional uses of camel have emerged including tourism, racing events and beauty contests, all emphasizing the fact that camel is a precious multipurpose animal (Faye, 2015). Several attempts have been carried out to understand the mystery of camel adaptation and the incredible capability of camel to withstand dehydration, thermal stress and other harsh environmental conditions (Ouajd and Kamel, 2009; Gebreyohanes and Assen, 2017). A distinctive coordination of anatomical, physiological and behavioral criteria is found to play a role in such efficient adaptation.

At the anatomical level, several adaptations have been identified: the one humped camel is provided with a single hump filled with fat rather than the common belief of being filled with water (Mohammed et al., 2005). The high fat content in camel humps serves as an energy store which is used in periods of food limitation (Chilliard, 1989). Camel nostrils have a muscular nature which allows camel to fully control its opening and closure, thus avoiding sand inhalation in case of sandstorm events (Gebreyohanes and Assen, 2017). The feet of camel are thick and characterized by leathery pads which spread widely on hitting the ground, consequently preventing the animal from sinking into the warm sand. Camel legs are long compared to other desert animals and during walking each two legs move on one side, rocking side-to-side, therefore giving another reason for being nicknamed the ship of desert. Among the interesting internal anatomical features observed in camels is the unique water sac structure in the stomach serving to store water (Allouch, 2016). Interestingly, the anatomical arrangement or distribution of camel arteries and veins help mitigate the high blood temperature of the body reaching the brain, thus protecting the animal from potential brain damage. This mechanism is referred as “selective brain cooling” (Ouajd and Kamel, 2009).

On the other hand, many physiological and behavioral aspects promote the acclimatization of Arabian camels to the extreme heat of the desert. For instance, there is another supporting mechanism to the previously mentioned selective brain cooling known as “adaptive heterothermy.” By this mechanism, camel can fluctuate its body temperature between 34 and 42°C, thus minimizing perspiration and avoiding water losses through evaporation (Ouajd and Kamel, 2009). Additionally, camels usually huddle together in order to cool themselves as their body temperature is often less than the surrounding air (Wilson, 1989). Moreover, in the recumbent position, the camel sternum takes a “plate like” conformation permitting more air circulation (Ouajd and Kamel, 2009). Furthermore, camel kidneys are able to efficiently excrete highly concentrated urine consequently tolerating high salt concentrations (Siebert and Macfarlane, 1971). Other physiological particularities include the capability of dromedary to drink huge amounts of water, reaching up to 200 liters at one time to compensate for fluid loss (Ouajd and Kamel, 2009). Red blood cells (RBCs) of camelids are anucleated with an exotic elliptical shape, to presumably facilitate their flow inside blood vessels in dehydrated animals (Al-Swailem et al., 2007; Warda et al., 2014). Moreover, camel platelets can resist high temperatures of 43–45°C which cause marked structural and functional alterations as compared to human platelets. Even higher thermal stress of 50°C that damages human platelets has slight effects on camel cells and does not critically disrupt their function (Al Ghumlas et al., 2008). Surprisingly, camel RBCs possess distinctive membrane phospholipid composition, resulting in a more fluid membrane, and enabling them to bear high osmotic variations without rupturing even in cases of rapid rehydration (Warda and Zeisig, 2000; Warda et al., 2014). Moreover, antibodies in *C. dromedarius* comprise dimeric heavy chains lacking the light chains, however, they display an extensive antigen-binding repertoire (Hamers-Casterman et al., 1993).

All these interesting facts denote further potential unrevealed mechanisms at the molecular level for camel adaptation to various stresses. Indeed, as partially presented in this section, various biomolecules and elements have been characterized in camelids and aided to increase our knowledge about their exceptional cellular homeostasis. However, in the current review we emphasize on camel HSPs as key players in its adaptation to heat stress.

## Classification of Family Camelidae

**FIGURE 1:**
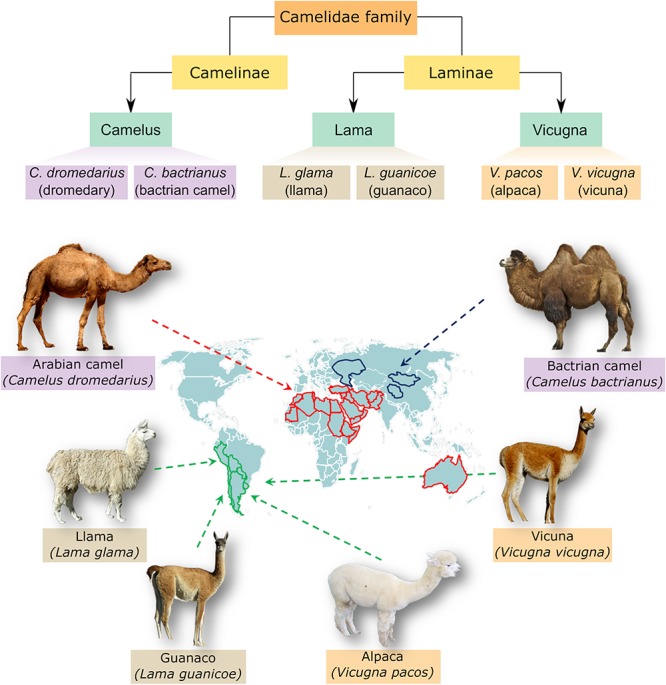
Classification and distribution of camel species. The upper panel demonstrates the genera and species belonging to camelidae family. These include three genera, *Camelus*, *Lama*, and *Vicugna*, which comprise large camel species like Arabian camel (one humped camel) and Bactrian camel (two humped camel) as well as small camelids like llama, alpaca, guanaco and vicuna. *Camelus ferus*, which is a double humped wild Bactrian camel, is not shown for simplicity. The lower panel shows a visual distribution map of different species within camelidae family.

To get a clearer picture about the organism under study, an obvious simplified classification is presented to avoid confusion with other species within the Camelidae family. The family of Camelidae comprises two major subfamilies, namely Camelinae (Old World Camelids) and Laminae (New World Camelids). The old world camelids include two domesticated species; the dromedary or one humped camel (*C. dromedarius*) and the two humped camel or bactrian camel (*C. bactrianus*). Both species are referred to as large camelids and distributed into different regions of the world. Arabian camel (*C. dromedarius*) is located mainly in the hot areas of Middle East and Africa whereas *C. bactrianus* inhabit the cold zones of Central Asia and China (Al-Swailem et al., 2007; Kadim et al., 2013). The new world camelids comprise four main species located in South America and are commonly known as small camelids. Yet, two species the llama (*Lama glama*) and the alpaca (*Vicugna pacos*) have been domesticated whereas the other two species, namely the guanaco (*L. guanicoe*) and the vicuna (*V. vicugna*) are wild species (Dorman, 1984; Kadim et al., 2008; Faye, 2015). A schematic classification and map distribution of members of the camelidae family is shown in Figure 1.

## Adaptation to Desert Conditions is Integrated in the Dromedary Genome

Consistent with their highlighted physiological and anatomical adaptation to desert conditions, dromedary camels have shown interesting findings at the genomic level. In a pioneering work by Wu et al. (2014), they did high quality genome sequencing of three camel species including; *C. dromedarius*, *C. bactrianus*, and alpaca (*V. pacos*). Data achieved through comparative genomic and transcriptomic analyses of those species indicated numerous features with high potential to desert adaptation. For example, the dromedary showed enhanced energy and fat metabolism, water reservation, salt metabolism, osmoregulation and sodium reabsorption. Moreover, stress related genes such as those involved in DNA damage and repair, apoptosis, protein stabilization and immune responses were found superior in terms of accelerated evolution compared to their homologs in cattle species (Wu et al., 2014). In another interesting study, the whole genome sequencing of Iranian dromedaries revealed genetic variations, including single-nucleotide polymorphisms (SNPs) and indels (insertions and deletions) between the compared species. However, the majority of genes associated with stress response were clearly identified in the species under study suggesting efficient adaptation of the Iranian dromedaries to the desert milieu of Iran. Identifying genetic variations including SNPs among native camel species represents a step forward to better understand camel evolution and improves camel breeding programs (Khalkhali-Evrigh et al., 2018).

## Heat Shock Proteins in Arabian Camel in Relation to Other Animals

As desert animals, Arabian camels are subjected to extended periods of heat stress which require efficient cellular and molecular buffers. Over the past decade, increasing interest toward camel HSPs has evolved to unravel their potential role in thermotolerance. Most of the studies focused on cloning and characterizing representative HSP members from camel tissues whereas other studies focused on the analysis of HSP expression either in living animals or in mammalian models. Here, we review the highlights of the all respective studies.

## HSPA Family (HSP70)

**FIGURE 2:**
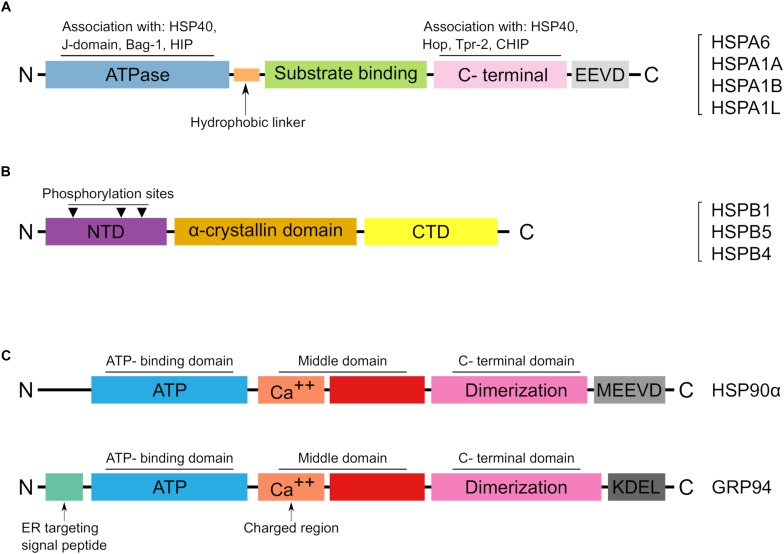
The main structural features of reported HSPs in Arabian camel. **(A)** Schematic representation of the HSP70 structural domains characterized from *Camelus dromedarius*. A list of identified HSP70 members in Arabian camel is shown on the right side. **(B)** The structural characteristics of HSPB proteins in Arabian camel. The reported protein sequences are highly homologous to their human peers and preserve the α-crystalline domain as well as the phosphorylation sites. **(C)** Structural topology of the reported HSP90 members in Arabian camel. The upper panel shows the cytoplasmic HSP90α while the lower panel reveals the ER localized GRP94. HSP90α differs from its ER paralog in lacking both the ER target and retention signals, instead it has a C-terminal MEEVD motif.

The HSP70 family comprises highly conserved proteins with molecular weight of 70 kDa which are largely known to resist heat as well as several stresses. In humans, the family HSP70 comprises thirteen members which share high sequence and structural homology, however, may vary or overlap in their functions (Daugaard et al., 2007; Kampinga et al., 2009; Table 1). The main structural features in HSP70 members include N-terminal domain which has an ATPase activity, a middle domain and a C-terminal domain (Figure 2A). The HSP70 family includes housekeeping or continuously expressed proteins in addition to other inducible members. Compared to other HSP families, HSP70 has been mostly studied in correlation to thermal and environmental stresses (Pyza et al., 1997; Ackerman et al., 2000; Kumar et al., 2003).

**TABLE 1:** Various members of HSP70 (Kampinga et al., 2009).

**Gene name**	**Protein name**	**Alternative name**	**Human Gene ID**
*HSPA1A*	HSPA1A	HSP70-1; HSP72; HSPA1	3303
*HSPA1B*	HSPA1B	HSP70-2	3304
*HSPA1L*	HSPA1L	hum70t; hum70t; Hsp-hom	3305
*HSPA2*	HSPA2	Heat-shock 70kD protein-2	3306
*HSPA5*	HSPA5	BiP; GRP78; MIF2	3309
*HSPA6*	HSPA6	Heat shock 70kD protein 6 (HSP70B’)	3310
*HSPA7*	HSPA7	Heat shock 70kD protein 7	3311
*HSPA8*	HSPA8	HSC70; HSC71; HSP71; HSP73	3312
*HSPA9*	HSPA9	GRP75; HSPA9B; MOT; MOT2; PBP74; mot-2	3313
*HSPA12A*	HSPA12A	FLJ13874; KIAA0417	259217
*HSPA12B*	HSPA12B	RP23-32L15.1; 2700081N06Rik	116835
*HSPA13b*	HSPA13b	Stch	6782
*HSPA14*	HSPA14	HSP70-4; HSP70L1; MGC131990	51182

Early studies on camel lymphocytes revealed high competence of general protein synthesis as compared to humans (Ulmasov et al., 1993). Also, the expression of HSP73 in camel erythrocytes did not show high levels of expression as compared to its expression in lymphocytes. Further molecular analysis of HSPs in camel lymphocytes revealed strong induction of HSP73 upon exposure to thermal stress, whereas the constitutively expressed HSP75 was not equally induced upon the same temperature exposure (Ulmasov et al., 1993).

Further studies on the camel fibroblast cell line model, Dubca, revealed an interesting phenomenon in terms of cell survival. Upon exposure to an elevated temperature of 42°C, the Dubca cells survived the heat stress conditions applied over 48 h and continued to grow while parallel murine fibroblast cells (L929) subjected to the same stress conditions were almost dead upon 24 h exposure (Thayyullathil et al., 2008). Furthermore, the recovery of Dubca cells occurred at a faster rate when compared to the murine cells. Surprisingly, when analyzed by western blot, the HSP70 expression in heat stressed Dubca cells was unexpectedly similar to normal physiological conditions; however, the study revealed the expression of an additional lower size isoform which was suggested to play a role in the thermotolerance of camel cells (Thayyullathil et al., 2008). Other protein levels of the prosurvival kinase Akt were not altered following 42°C heat stress while the c-Jnk expression was diminished in accordance with the fact that downregulation of c-Jnk has been associated with enhanced thermotolerance (Thayyullathil et al., 2008).

Garbuz et al. (2011) succeeded in mapping a genomic cluster comprising three genes of HSP70 family in *C. dromedarius* namely, *HSPA1A*, *HSPA1L*, and *HSPA1B*. These genes were closely associated with the major histocompatibility complex (MHC) class III region. Two mapped HSP70 genes contained heat shock elements (HSEs) required for heat shock induction, while the third one lacked any (HSEs) sequences. When compared with the corresponding loci in other animals, the camel HSP70 cluster appeared highly conserved and kept similar organization to other species. Notably, the two heat inducible HSPs were tandemly arranged whereas the third constitutive HSP70 gene was located in a reverse orientation. On the other hand, comparison of the regulatory sequences of HSP70 genes in camel and other mammals revealed a greatly conserved arrangement, whereby the sites of transcription factors were localized 250 bp upstream region followed by *NF-Y* and *Sp1* binding sites. Interestingly, the three HSP70 genes were expressed in blood and muscle of camel under both physiological and heat stress conditions indicating a potential thermotolerant role. Overall, the high conservation of HSP70 arrangement in close association with *MHC* locus pinpoints a coordinated functioning of these crucial genes (Garbuz et al., 2011).

Among the HSP70 members, camel HSPA6 attracted a lot of interest. This inducible HSP is strictly regulated, and its expression is reported to be increased after severe cellular stress (Noonan et al., 2007). Interestingly, isolated cDNA of camel HSPA6 showed sequence differences between the cDNA isolated from Saudi Arabia and that isolated from Egypt suggesting the existence of natural variants of HSP70 members among Arabian camel strains (Elrobh et al., 2011; Hoter et al., 2018a). Moreover, in-depth molecular investigation of camel HSPA6 (cHSPA6) revealed that the expression of its isoforms in mammalian cells varies from those exhibited by the human HSPA6 (hHSPA6) homolog (Hoter et al., 2018a). For instance, comparative SDS-PAGE analysis of HSPA6 showed two main isoforms of cHSPA6 while hHSPA6 exhibited 3 closely spaced isoforms at the size level of 70 kDa. The differential mobility pattern on SDS-PAGE suggested differential cellular processing of HSPA6 in the two species. Interestingly, cHSPA6 appeared to contain two-fold increase in *O*-GlcNAc binding sites compared to the human homolog. Further supportive experiments indicated that, unlike the human ortholog, the upper cHSPA6 isoform was comparatively hyperglycosylated and this *O*-glycosylation happens in non-stressed cells as well as hypoxic and heat-stressed cells. The fact that *O*-glycosylation of cytosolic proteins including HSPs promotes stress resistance (Zachara and Hart, 2004) confers additional value to HSPA6 in tolerating heat stress in Arabian camels (Hoter et al., 2018a).

In another interesting study, Sadder et al. (2015) evaluated the HSP expression profiles in Arabian camels under controlled environmental stress. The animals selected were maintained in a constrained climatic chamber and were heat challenged by exposure to 43°C for different time points. HSP expression levels estimated from the collected blood revealed a sharp increase in the mRNA levels of HSP60, HSPA6, HSP105, HSP70, and HSPA1L after 3 h of heat challenge followed by decreased levels after 6 h. Interestingly, the expression of camel HSPs including HSPA6 rebounded after 24 h of heat stress (Sadder et al., 2015). Similarly, the fluctuation of HSP70 expression levels has been reported in cattle where the HSP70 gene expression revealed an initial increase in expression within 1–2 h, followed by a down-regulation after 8 h (Collier et al., 2006; Sadder et al., 2015). In fact, tracking the expression of HSPs under stress conditions in variant animals reflects an outline about the cellular fate in terms of apoptosis/death, or alternatively, stress resistance and cell survival. Also, linking the HSP expression data with animal performance and productivity would help in genetic selection of heat resistant phenotypes (Sadder et al., 2015).

Other recent studies performed on camel cells showed differential tolerance to heat stress (Saadeldin et al., 2018). Both camel oocytes and cumulus granulosa cells were exposed to high temperature of 45°C for 2 or 20 h, representing acute and chronic heat stress conditions. Camel oocytes revealed lower resistance to the applied acute thermal stress unlike cumulus cells which tolerated both acute and chronic stresses. Remarkably, the analysis of mRNA transcripts in both cell types demonstrated significant increases in the expression of *HSP70* and *HSP90* in cumulus compared to their expression in oocytes, indicating that the induction of HSP70 and HSP90 contributes to the preferential enhanced tolerance of cumulus cells to either acute or chronic stress (Saadeldin et al., 2018).

## HSPB Family (Small Heat Shock Proteins, sHSPs)

Small heat shock proteins (sHSPs) are chaperones of small molecular weight, ranging from 12 to 43 kDa. These molecules play a key role in cellular stress resistance and are widely expressed in many cell and tissue types (Bakthisaran et al., 2015). According to their distribution in variant tissues, sHSPs have been classified into two classes: class I and class II (Taylor and Benjamin, 2005). Class I sHSPs includes proteins of ubiquitous expression in almost all tissue types like HSPB1, HSPB5, HSPB6, and HSPB8 while class II sHSPs includes members of target tissue distribution such as HSPB2, HSPB3, CRYAA, HSPB7, HSPB9, and HSPB10 (Taylor and Benjamin, 2005). sHSPs are distinguished from other large molecular weight HSPs by their ATP-independent activity (Basha et al., 2012). Structurally, members of sHSPs share the highly conserved “α-crystallin domain” (ACD) which is considered the hallmark of sHSPs (Basha et al., 2012; Figure 2B). A recent classification of human HSPs has designated the name HSPB for sHSP members as shown in Table 2 (Kampinga et al., 2009).

**TABLE 2:** Members of HSPB (small heat shock proteins) family (Kampinga et al., 2009).

**Gene name**	**Protein name**	**Alternative name**	**Human Gene ID**
*HSPB1*	HSPB1	CMT2F; HMN2B; HSP27; HSP28; HSP25; HS.76067; DKFZp586P1322	3315
*HSPB2*	HSPB2	MKBP; HSP27; Hs.78846; LOH11CR1K; MGC133245	3316
*HSPB3*	HSPB3	HSPL27	8988
*HSPB4*	HSPB4	crystallin alpha A; CRYAA, CRYA1	1409
*HSPB5*	HSPB5	crystallin alpha B, CRYAB; CRYA2	1410
*HSPB6*	HSPB6	HSP20; FLJ32389	126393
*HSPB7*	HSPB7	cvHSP; FLJ32733; DKFZp779D0968	27129
*HSPB8*	HSPB8	H11; HMN2; CMT2L; DHMN2; E2IG1; HMN2A; HSP22	26353
*HSPB9*	HSPB9	FLJ27437	94086
*HSPB10*	HSPB10	ODF1; ODF; RT7; ODF2; ODFP; SODF; ODF27; ODFPG; ODFPGA; ODFPGB; MGC129928; MGC129929	4956
*HSPB11*	HSPB11	HSP16.2; C1orf41; PP25	51668

So far, two members within sHSPs family have been identified in Arabian camel; HSPB5 or (αB-crystallin, CRYAB) and HSPB1 or HSP27 (Manee et al., 2017; Hoter et al., 2018a). Molecular investigations of camel CRYAB showed that its coding cDNA contains 528 bp encoding a protein of 175 amino acid residues. Expression analysis of the recombinant cCRYAB by SDS-PAGE reflected a protein band with molecular mass of 25 kDa while confocal microscopic examination of the expressed protein revealed dominant cytoplasmic localization (Hoter et al., 2018a). The cDNA of camel CRYAB and its deduced amino acid sequence showed high similarity and identity with human and other animals. Moreover, camel CRYAB possess the conservative alpha crystalline domain and the putative phosphorylation sites at Ser19, Ser45, and Ser59 (Warda et al., 2014; Hoter et al., 2018a). Phosphorylation of these sites by the MAPK kinase MKK6 potentiates the cytoprotective and chaperone activity of CRYAB in terms of counteracting stress, induced protein aggregation and stabilization of partially denatured or misfolded proteins (Hoover et al., 2000; Bakthisaran et al., 2016).

On the other hand, the camel HSPB1 (HSP27) has a cDNA with open reading frame (ORF) of 606 bp which encodes a protein of 201 amino acids (Manee et al., 2017). The mRNA expression levels of *HSPB1* showed ubiquitous, however, differential expression in various tissues. In non-stressed conditions, the examined camel tissues showed the highest expression of *HSPB1* mRNA in esophagus, skin, and heart compared to the lowest expression in the brain, spleen, and stomach tissues (Manee et al., 2017). When heat stressed for long time at 42°C, the camel skin fibroblast cells (SACAS) showed notable upregulation of HSPB1 following 6 h of incubation compared to control cells incubated at 37°C. These findings indicate that induced expression of sHSPs in Arabian camel is both tissue specific and time dependent (Manee et al., 2017).

Warda et al. (2014) performed an interesting comparative study of camel and rat proteomes in multiple tissues. The comparative proteomic analysis demonstrated marked overexpression of camel CRYAB in camel heart with seven-fold increase as compared to rat heart. This relative increase in cardiac CRYAB expression can be explained considering- the high cytoprotective demand and protein anti-aggregative activity offered by the ATP-independent chaperone. As a consequence, camel heart can utilize minimum energy to withstand the risk of stress induced protein misfolding or aggregation (Warda et al., 2014). Another fruitful outcome from the abundant CRYAB expression in camel heart is promoting structural integrity and providing extra protective roles against dehydration and sudden rehydration in the harsh desert milieu (Warda et al., 2014). Further proteomic analysis of Arabian camel hump revealed excess array of adipose tissue associated cytoskeletal proteins such as actin, tubulin and vimentin in addition to heat shock proteins including HSP27 and HSP70. These cytoskeletal proteins and heat shock proteins provide structural integrity and ensure efficient heat stress tolerance and general protein homeostasis (Warda et al., 2014).

## HSPC (HSP90 Family)

HSP90 family is a class of HSPs that has an estimated molecular weight of 90 kDa (Csermely et al., 1998; Chen et al., 2005). This family comprises four major members which are well conserved in higher eukaryotic species; these include HSP90α, HSP90β, GRP94, and TRAP1. HSP90 family has been recently named HSPC according to the guidelines of the HUGO Gene Nomenclature Committee (HGNC) (Kampinga et al., 2009) as presented in Table 3, however, they are still eminent with the old name HSP90. Due to their importance and implication in various cellular as well as pathological events (Hoter et al., 2018c), HSP90 isoforms are distributed in crucial cellular compartments. For instance, the two members HSP90α/β are localized in the cytoplasm (Li and Buchner, 2012; Schopf et al., 2017), GRP94 resides in the endoplasmic reticulum (Yang and Li, 2005) and TRAP1 has a mitochondrial preference (Altieri et al., 2012). The vital physiological processes maintained by HSP90 members include protein folding, cell proliferation and differentiation, apoptotic processes, hormone signaling and cell cycle control (Csermely et al., 1998; Hoter et al., 2018c). Additionally, HSP90 candidates are linked to many pathologies such as cancer, inflammation and neurodegenerative diseases (Whitesell and Lindquist, 2005; Luo et al., 2010; Sevin et al., 2015).

**TABLE 3:** Different candidates of HSP90 family (Kampinga et al., 2009).

**Gene name**	**Protein name**	**Alternative name**	**Human Gene ID**
*HSPC1*	HSPC1	HSP90AA1; HSPN; LAP2; HSP86; HSPC1; HSPCA; HSP89; HSP90;HSP90A; HSP90N; HSPCAL1; HSPCAL4; FLJ31884	3320
*HSPC2*	HSPC2	HSP90AA2; HSPCA; HSPCAL3; HSP90ALPHA	3324
*HSPC3*	HSPC3	HSP90AB1; HSPC2; HSPCB; D6S182; HSP90B; FLJ26984; HSP90-BETA	3326
*HSPC4*	HSPC4	HSP90B1;ECGP; GP96; TRA1; GRP94; endoplasmin	7184
*HSPC5*	HSPC5	TRAP1; HSP75; HSP90L	10131

In *C. dromedarius*, two candidates of HSP90 have been characterized on a molecular level. The first member that was characterized is HSP90α (Saeed et al., 2015). This cytoplasmic chaperone is induced by variant stresses and is considered as the major form (Sreedhar et al., 2004). Although it displays a high homology at the protein level with its cytoplasmic constitutive homolog, HSP90β, there exists functional differences between the two HSP90 isoforms (Sreedhar et al., 2004; Hoter et al., 2018c). The coding cDNA of camel HSP90α encodes a protein of 733 amino acid residues and shares high similarity and identity with other mammalian HSP90α. Comparative protein sequence analysis reveals over 85% identity between camel and other animals including cattle, horse, dog, cat and human (Saeed et al., 2015). Also, the structural architecture of HSP90 family has been well preserved in the camel HSP90α; these include the N-terminal domain, the middle domain and C-terminal domains with its peptide MEEVD motif. The main structural features of camel HSP90 is demonstrated in Figure 2C.

The second HSP90 member characterized in Arabian camel is endoplasmin or glucose regulated protein, GRP94 (Hoter et al., 2018b). This ER resident chaperone is an essential member of the ER quality control machinery. It helps protein folding of nascent polypeptides, binds calcium by its calcium binding domain, interacts with other ER chaperones and targets misfolded proteins to the ER associated degradation pathway (ERAD) (Marzec et al., 2012). Interestingly, camel endoplasmin shares 100% protein identity with that of human and more than 98% with other close mammals (Hoter et al., 2018b). As a consequence, the structural characteristics and posttranslational modifications of the ER chaperone resemble those in human. For instance, camel GRP94 contains a signal sequence at its N-terminal, comprising the first 21 amino acid residues which is cleaved co-translationally to give the mature form of the protein. Also, cGRP94 contains the classical domains: N-terminal domain (NTD), acidic linker domain (LD), middle do- main (MD) and the C- terminal domain (CTD) besides the well-known ER retention motif KDEL (Hoter et al., 2018b). The high protein and structural conservation of HSP90 members among mammals including camel is interesting and indicates the global significance of these HSPs in higher eukaryotes.

## Conclusion and Future Perspectives

For a long time, the Arabian camel has been appreciated as a mean of transport in the desert and as a source of food in terms of meat and milk. Despite the conditions of drought, food limitations and extreme temperatures these interesting creatures can reproduce and function normally without any physiological impairment, a phenomenon that attracts our scientific curiosity to investigate and decipher. Though valuable effort has been done to elucidate the physiological and biochemical secrets of Arabian camel, more in-depth investigations of the molecular adaptation mechanisms in these animals are needed. HSPs, as critical elements in stress response and thermotolerance particularly in desert animals, are worthy candidates to study and evaluate. In this regard, we reviewed the current knowledge in the field of camel HSPs and we strongly encourage further molecular studies of other undeciphered members. Molecular studies of camel HSPs would help expand our knowledge about HSPs and consolidate the interesting physiological phenomena in Arabian camel.

## Author Contributions

AH and HN conceived the review topic. AH wrote the first draft and designed the figures. SR and HN edited and approved the final version of the review.

## Conflict of Interest Statement

The authors declare that the research was conducted in the absence of any commercial or financial relationships that could be construed as a potential conflict of interest. The handling Editor and reviewer PO-t declared their involvement as co-editors in the Research Topic, and confirm the absence of any other collaboration.
